# Exploring Trade-Offs Between Profit, Yield, and the Environmental Footprint of Potential Nitrogen Fertilizer Regulations in the US Midwest

**DOI:** 10.3389/fpls.2022.852116

**Published:** 2022-04-15

**Authors:** German Mandrini, Cameron Mark Pittelkow, Sotirios Archontoulis, David Kanter, Nicolas F. Martin

**Affiliations:** ^1^Department of Crop Sciences, University of Illinois at Urbana-Champaign, Champaign, IL, United States; ^2^Department of Plant Sciences, University of California, Davis, Davis, CA, United States; ^3^Department of Agronomy, Iowa State University, Ames, IA, United States; ^4^Department of Environmental Studies, New York University, New York, NY, United States

**Keywords:** environmental policy, bio-economic modeling, externalities, nitrogen pollution, nitrogen use efficiency

## Abstract

Multiple strategies are available that could reduce nitrogen (N) fertilizer use in agricultural systems, ranging from voluntary adoption of new N management practices by farmers to government regulations. However, these strategies have different economic and political costs, and their relative effectiveness in decreasing N leaching has not been evaluated at scale, particularly concerning potential trade-offs in crop yield and profitability. To inform policy efforts in the US Midwest, we quantified the effects of four policy scenarios designed to reduce fertilizer N inputs without sacrificing maize yields below 95%. A simulated dataset for economically optimum N rates and corresponding leaching losses was developed using a process-based crop model across 4,030 fields over 30 years. Policy scenarios were (1) higher N prices, (2) N leaching fee, (3) N balance fee, and (4) voluntary reduction of N use by farmers, each implemented under a range of sub-levels (low to high severity). Aggregated results show that all policies decreased N rates and N leaching, but this was associated with an exponential increase in economic costs. Achieving an N leaching reduction target of 20% has an estimated pollution control cost of 30–37 US$/ha, representing 147 million US$/year when scaled up to the state level, which is in the range of current government payments for existing conservation programs. Notably, such control of N losses would reduce the environmental impact of agriculture on water quality (externalities) by an estimated 524 million US$/year, representing an increase in society welfare of 377 million US$/year. Among the four policies, directly charging a fee on N leaching helped mitigate economic losses while improving the point source reduction effect (i.e., targeting fields that were leaching hotspots) and better internalization effect (i.e., targeting fields with higher environmental impact costs). This study provides actionable data to inform the development of cost-effective N fertilizer regulations by integrating changes in crop productivity and N losses in economic terms at the field level.

## Highlights

- Policy instruments can be used to induce reductions in N fertilizer use (19 %) and N leaching (20%) with minimal effect on yield (<3%)
- A reasonable target is a 20% N leaching reduction, which resulted in a social cost of pollution control of 34 US$/ha (147 million US$/year at the state level)
- Such N leaching reduction would reduce externalities by 524 million US$/year, resulting in a social return on investment of 260%
- The N leaching fee policy was slightly more cost-efficient, reducing N leaching more in fields with high N leaching (controlling hotspots)

## 1. Introduction

Nutrient pollution in the United States (US) Midwest is a challenging sustainability issue facing modern agriculture. One of the main externalities of excessive N losses from agricultural fields is environmental and health damages estimated at US$ 157 billion per year (Sobota et al., 2015). The Hypoxia Task Force (HTF) is a federal-state partnership established in 1997 to reduce nutrient concentrations in the Midwest's main rivers and reduce the size of the hypoxic zone in the Gulf of Mexico. The HTF delivered an action plan to Congress, intending to reduce the dead zone's 5-year average areal extent to less than 5,000 km^2^, which would require a 45% reduction in river line total nitrogen load (HTF, 2017). Aligned with those federal efforts, the Illinois Nutrient Loss Reduction Strategy (NLRS) was developed at the state level to identify existing and proposed actions that may contribute to nutrient load reductions. Both federal and state governments have avoided direct regulation of agriculture. Instead, they have relied on voluntary and incentive-based policy tools (Reimer et al., 2018). Despite current efforts, the hypoxic zone's areal extent is much larger than the HTF goal, and river nitrate concentrations have not declined since the 1980's (Sprague et al., 2011; Murphy et al., 2013).

Innovative policies are needed to advance sustainability (Shortle et al., 2012; Khanna et al., 2019; Kanter et al., 2020; Spangler et al., 2020), benefiting not only this region but serving as an example for other global breadbaskets. One way of achieving reductions is to use economic instruments that induce farmers to use less N fertilizer, below the N rates that maximize profits under current market conditions. However, to be effective, such policies must integrate both a strong understanding of factors controlling crop productivity and N losses at the field level, where N management decisions are made, as well as the economic consequences for farmers and regional scale environmental consequences. We identified four main options that could potentially be implemented in the region of study to induce lower N rates, which include: (1) modification of the N:maize price ratio (Sheriff, 2005; Wu and Tanaka, 2005; Finger, 2012; Zhang et al., 2015b), (2) imposing fees to the amount of N leaching occurring in specific fields (Horner, 1975), (3) imposing fees on N balance indicators that are indices expected to represent the surplus of nitrogen on agricultural land (Walker and Swanson, 1974; Fried et al., 1976; Martinez-Feria et al., 2018), and (4) promote voluntary reductions in N use by farmers (Swanson, 1982).

While each policy has strong potential for reducing N losses, few studies have directly compared their economic and environmental impacts, leaving unanswered questions about their relative effectiveness and regional-scale consequences. Notably, the mechanism of each policy targets different indicators (i.e., economic cost of fertilizer, N losses, the efficiency of N use), meaning differences will exist in the political implications and feasibility of implementation. To our knowledge, a cost-benefit analysis of policies that would induce farmers to use less N fertilizer has not been conducted, considering both changes in N leaching and trade-offs in crop yield and profitability using field-level data in this region, generating a major knowledge gap. Addressing these questions is a core issue for developing targeted policies that make efficient use of public conservation dollars while protecting the economic interests of farmers and increasing the eco-efficiency of food production systems.

One reason for the knowledge gap is that yield and N leaching responses to N fertilizer are highly variable in time and space due to complex interactions between soil, weather, and management (Tremblay et al., 2012; Ransom et al., 2020). Hence, any reasonable evaluation of different policy scenarios should consider multiple years of results over a wide range of biophysical conditions. However, such studies have a high economic cost and face the challenge of being sustained over a long period.

Cropping system simulation models can be used to generate data and compensate for the lack of long-term field data (Basche et al., 2016; Puntel et al., 2016; Sela et al., 2018). Here, we used a dataset simulated using the Agricultural Production Systems sIMulator (APSIM) (Holzworth et al., 2014) for 4270 fields in the state of Illinois, one of the largest contributors of N leaching from the Midwest (Illinois-EPA, 2015). These models can generate information at a scale not possible with field experiments, providing a powerful tool to explore pathways for reducing N losses at the regional level. The strength of our field-level modeling approach relies on first quantifying relationships between economic optimum N rates (EONR) and N leaching losses under highly variable soil and climate conditions and then upscaling results to provide insights about the net impacts of different policies for decision-makers.

Despite decades of research and publicly funded conservation programs, little progress has been made in reducing N losses (Environmental Protection Agency, 2000; Gilinsky et al., 2009; Ribaudo, 2009), and climate change is expected to exacerbate the problem. While there are increasing calls for actions that help reduce N loss from agricultural systems while maintaining farmers' profitability (Khanna et al., 2019), the effects of different policies remain poorly understood. The objectives of this study were to: (i) evaluate the impact of the identified four policies on N fertilizer use, N leaching, and economic returns compared to the no-policy situation, (ii) compare the effects of policies at the field level in terms of their capacity to maintain income relative to the no-policy situation (income effect), their ability to penalize fields with high N leaching more precisely (internalization effect), and their capacity to target and control hotspots of N leaching (point source reduction effect). We focused only on these four policies and made specific assumptions on how they could be implemented (i.e., thresholds above which the fees are charged or how compensation is paid to farmers to avoid income reductions). Model simulations have limitations and results are meant to serve as an example in discussing potential policies and how they should be implemented, providing decision-makers with actionable data to assess the relative risks and cost of mitigating N leaching.

## 2. Methods

### 2.1. Simulations Description and Site Characterization

This work used the calibrated and validated dataset presented in detail in Mandrini et al. (2022). In brief, the data consisted of simulations for 4,270 fields, run using APSIM. The fields had a maize-soy rotation during 1989–2018 (30 years). Each time a field was assigned to maize, the response to increasing N rates from 0 to 320 kg/ha was simulated. After a calibration and validation process, the model was able to reproduce known regional yield responses to N fertilizer and EONR variability. The validation also showed that the simulated N leaching area-weighted averaged for all the fields was correlated with real nitrate flow measurements in the Mississippi river, suggesting that sub-root nitrate losses are closely correlated with the nitrate concentrations of water bodies. We aggregated the data at the field level, and a description of the final database is provided in Supplementary Table S1.

The dataset represents current agricultural practices in Illinois, dominated by cropland planted to maize and soybeans (60 percent of the state's land area) and where most of N applied to crops is provided by synthetic fertilizers. Animal husbandry in open fields and manure applications is low in most of Illinois (Illinois-EPA, 2015), and this source of N was not accounted for in our analysis.

### 2.2. General Flowchart

This article combined crop modeling with economic analysis to identify economically optimum N rates and the corresponding effects on N leaching under different policy scenarios. Similar frameworks have proven to be useful for this type of research (Semaan et al., 2007; Kuhn et al., 2010; Finger, 2012).

**Figure 1:**
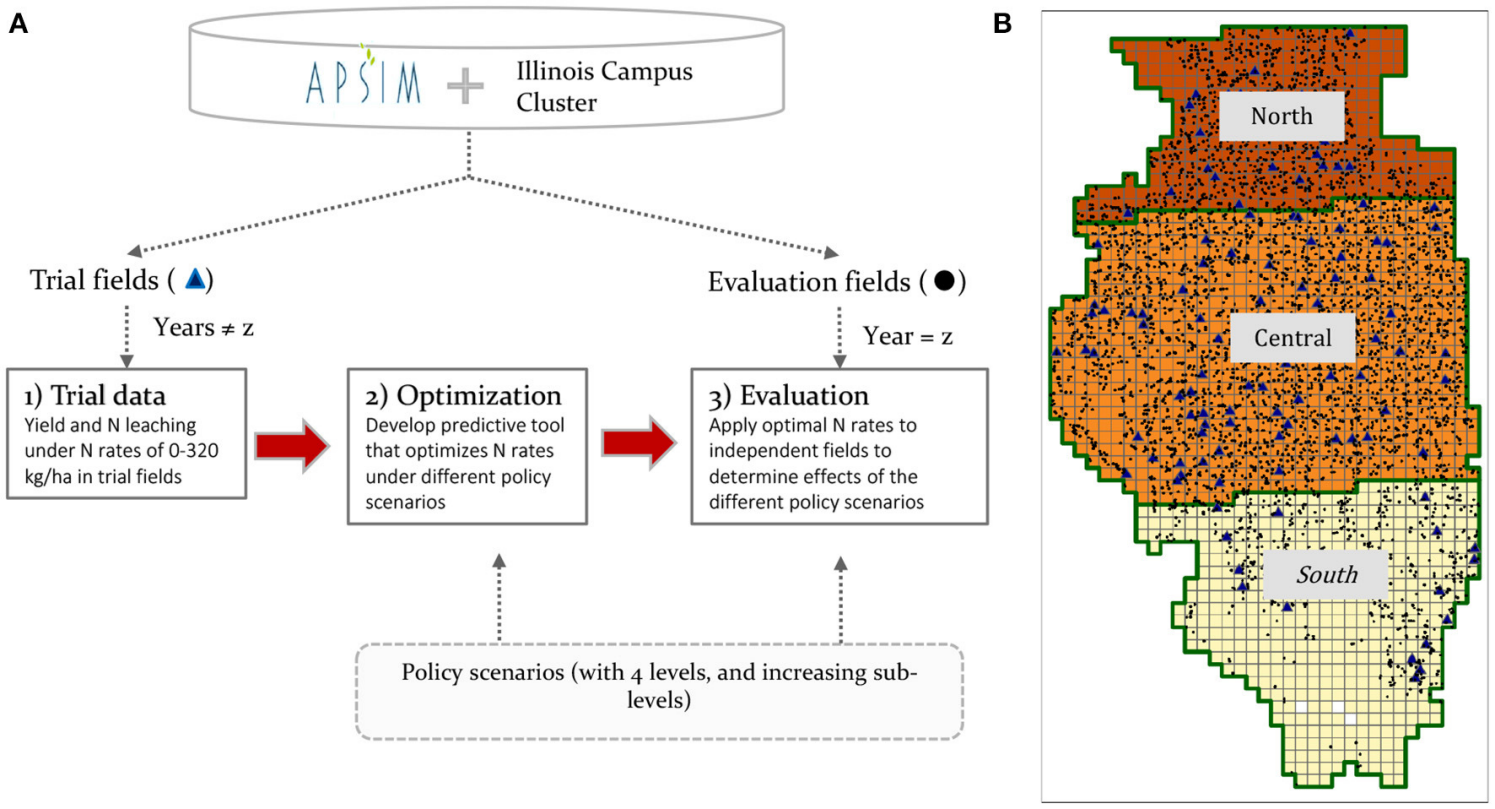
**(A)** Schematic diagram of the flow chart followed in the analysis. Boxes indicate the major processes. Arrows indicate the flow of information. **(B)** Map of Illinois, showing the grid of 10 x 10 km cells, the three regions, the trial fields (triangles), and the evaluation fields (dots).

A flowchart of the research pipeline is presented in Figure 1A, with specific details given below in different sections of the methodology. Our process followed a leave-one-year-out approach together with the partition of the fields into 240 trial fields and 4,030 evaluation fields (Figure 1B). For each year in the 30-year-long sequence, three stages were followed: (1) The information from trial fields of crop N response and N leaching for all the years except the one evaluated was gathered, (2) these responses were used to optimize an N recommendation tool that predicts the optimal N rates, adjusting for different policy scenarios, and (3) the N recommendation tool was used on the evaluation fields during the year that was left out from stage 1, to simulate impacts on yield and N leaching. In summary, the trial fields were the same in different years, and the performance of optimal N recommendations was evaluated in other fields called evaluation fields and in different weather years by using the leave-one-year-out approach.

### 2.3. Stage 1: Trial Data

For each year in the leave-one-year-out approach, the data from the trial fields for all the years, except the one left out, were used. The data consisted of 240 trial fields of maize response to multiple N rates (from 0 to 320 kg/ha, with 10 kg/ha increments). The variables measured on each trial were yield and N leaching, together with multiple soil, weather, and crop variables (Supplementary Table S1).

Because the trial fields also followed a maize-soy rotation, half of them provided N response curves on odd-numbered years and the other half on even-numbered years. In total, the trial data consisted of 3,480 N response curves, covering 29 weather years (120 fields x 29 weather years = 3,480 N response curves).

### 2.4. Stage 2: Optimization Module

The EONR varies across fields and years. Farmers need to make predictions early in the season, with incomplete information about the growing conditions during the season. For that, they need tools that will predict the EONR under specific soil and weather conditions.

In previous work, Mandrini et al. (2021) compared several N recommendation tools. They identified one called “rf_full” as the best tool to provide N recommendations when the crop had five expanded leaves (maize stage of v5), which is the time when N was applied. This tool utilized soil information (surface residue at v5, soil N at v5 0–60 cm, extractable soil water at v5, water holding capacity, soil organic matter at v5, sand, clay), weather information (rain, temperature, and radiation up to v5), crop information (long term yield, leaf area index at v5), and a technique called “random forest” to predict the EONR. We adopted this tool because it was the one that could predict EONR ex-ante (i.e., using information before v5) with the highest accuracy (measured as mean error and root mean squared error).

The random forest regressor was trained using the trial data from stage 1. For this, the EONR on each trial was selected. The EONR depended on the conditions set by the policy, as explained below. On the final training dataset, each row was a trial (field x year), the response variable was the EONR, and the predictor variables included early-season soil and weather conditions (Supplementary Table S1).

#### 2.4.1. Policy Scenarios

We evaluated four different policy scenarios that would induce farmers to use lower N rates: (I) The modification of the N:maize price ratio, (II) a fee on the N leaching from each field, (III) a fee on the N balance achieved by farmers, and (IV) a voluntary reduction of N application rates. For all the policies, we tested increasing sub-levels, which were increasing N price, leaching fee, N balance fee, or reduction targets, depending on the policy.

We acknowledge that there are management practices that reduce N leaching by means other than reducing N fertilizer inputs. This group includes N management practices, such as enhanced efficiency fertilizers (Kanter et al., 2015; Zhang et al., 2015b; Pittelkow et al., 2017) and timing of N applications (Kanter et al., 2015; Zhang et al., 2015b; Pittelkow et al., 2017; Banger et al., 2018; Ruffatti et al., 2019), other in-field management practices to capture and recycle N such as cover crops (Ruffo et al., 2004; Kling et al., 2014; Malone et al., 2014; Ruffatti et al., 2019), and edge-of-field practices to prevent N from moving into freshwater ecosystems such as bioreactors (Addy et al., 2016). However, there is less certainty about the magnitude and variability of N losses under these practices, and in some cases, they are harder to implement and adopt by farmers. Thus our scope was focused on modifying the N rate as a primary driver of N losses.

We established a base-level situation that reflects the current political and economic environment to determine the effects of the policies. For our simulations, this base-level situation included farmers applying the EONR recommended by rf_full with a price ratio of five (price of maize = 0.158 US$ kg^-1^, price of N = 0.79 US$ kgN^-1^), no fee applied for N leaching or N balance, and no voluntary reduction of N rates. This was considered the scenario in the area without policy intervention.

Not all farmers in the region used a random forest algorithm to predict the EONR, some of them use methodologies such as MRTN, and others used even higher N rates that guarantee that their crops will not be limited by N fertilizer. In those situations, we believe that policies will affect farmers using different tools to predict the EONR similarly (i.e., we assumed there was no policy x N recommendation tool interaction). In other words, if a farmer usually over-applies N, a policy that induces N fertilizer reductions will lower their N rate in a similar proportion than a farmer that is usually closer to the EONR. In consequence, the chosen method to predict EONR does not affect the estimated impact of the policies, as far as it is the same for the base-level situation and across the policies.

**I) N:maize price ratio modification**

We evaluated how increasing the price ratio between N and maize would affect both tools' profits and the dynamic value. The N:maize price ratio was defined as the ratio of the price per kilogram of N to the price per kilogram of maize. The equation for calculating the price ratio was:


(1) $$\begin{array}{ll} & Price\;ratio\;(kg\;maize/kg\;N)\\ & \;=\frac{Price\;of\;nitrogen\;fertilizer(US\$/kgN)}{Price\;of\;maize(US\$/kg\;maize)} \end{array}$$

An N:maize price ratio modification could be attained by increasing N's price or decreasing maize's price, achieving the same results. Here, we assumed a tax that modified N's price while keeping the maize's price constant.

The historical price ratio can be seen in Supplementary Figure S1. The current price ratio was set to 5 kg maize/kg N, and we tested sub-levels of this policy by increasing the price ratio to 20 kg maize/kg N.

**II) Leaching Fee**

This policy consisted of charging a fee to farmers for the “extra” N leaching generated in their fields, considering both maize and soybean years. Leaching is not only caused by N fertilizer but also by the mineralization of soil organic matter. Thus, base-level leaching increases when moving south to north due to the soils' higher organic matter. Considering that, we set a threshold by region, consisting of 60% of the 30-year average N leaching at the base-level situation (resulting in south = 18, central = 23, north = 29 kg N/ha). A fee was charged for N leaching above that threshold, inducing farmers to lower their N rates until the additional profit generated through higher maize yields was equivalent to the additional cost of higher N leaching under higher N rates. The policy sub-levels were created by increasing the fee from 0 to 40 US$/kg N ha.

Charging a fee only for the “extra” N leaching decreases the amount of the funds collected from farmers compared to setting a fee for all leaching generated on a field. This lowers the administrative effort while simultaneously producing the desired effect of reducing N rates. Also, identifying a different threshold for each region allows for distributing the policy's impact more equally across the state.

**III) Balance fee**

For this policy, we calculated the N balance as in Eagle et al. (2020), using:


(2) $$\begin{array}{r} Nbalance=Nfertilizer\left(kgN/ha\right)-Nremoved\left(kgN/ha\right) \end{array}$$

Where *Nfertilizer* is the N applied as mineral fertilizer, and *Nremoved* is the N harvested in maize grain (calculated from crop yield and an estimated grain N concentration of 11.5 g N/kg grain Tenorio et al., 2019)

The mean base-level N balance changes by region, decreasing from south to north, due to the lower N rates and higher yields observed in the mentioned direction. Like the previous policy, we set a threshold by region. After experimenting with several values, the best results were obtained when the threshold was calculated by subtracting 60 kg/ha to the 30-year average N balance at the base-level situation (resulting in south = 11, central = −23, north = −55 kg N/ha). An increasing fee (0 to 4 US$/kg N ha) was charged for the balance above that threshold.

Literature suggests that a positive N balance is desirable to avoid soil N mining and soil organic matter to decline (Zhang et al., 2015a; Zhao et al., 2016; McLellan et al., 2018; Quemada et al., 2020). Some of our thresholds were negative, and the reason is a natural contrast among the regions. The north had higher yields and lower N rates, which led to a low N balance. On the contrary, the south had lower yields and higher N rates, leading to a high N balance. Nevertheless, the north had higher N leaching than the south (Supplementary Figure S3). Setting an N balance threshold of zero will have a minor influence on the north's N rate decision, while it will force farmers in the south to lower their N rates. This will produce a perverse effect among regions, especially considering that farmers in the south are less profitable for precisely the same reasons that make their N balance high: low yields and high fertilizer cost. The chosen thresholds allow the policy to influence all regions similarly. Our work is not trying to get farmers to achieve those selected thresholds but rather inducing them to lower their N rates. The final N rate depends on the interaction of the fee and the threshold (i.e., a high fee will force farmers to apply N rates that lead to an N balance closer to the threshold, while a low fee will have almost no influence on farmers' N rate decision).

**IV) Voluntary Reduction**

In this policy scenario, farmers were assumed to voluntarily reduce their N rate, by a certain percentage, from the N rate predicted to maximize profits. For this, they reduced their N rate by increasing percentages (from 0 to 30%) from the N rate recommended at the base-level situation. We included the proposed policy to evaluate if farmers would be better off by voluntarily reducing their N rates in exchange for an incentive compensation equal to the economic loss, which is explained later. This policy is aligned with current efforts in the US Midwest, where most nutrient reduction strategies rely on voluntary action (HTF, 2017). Contrary to the previous policies, this policy does not involve any transfer of funds from farmers to the government.

#### 2.4.2. Optimization Process

Since policies targeted different components of profit calculations, the optimization process followed specific steps, explained in Supplementary Table S2 and equations provided in Supplementary Table S3. A new N rate recommendation tool was trained for each policy level and sub-level, considering the additional cost associated with higher fertilizer prices or fees charged for N leaching or N balance above the established thresholds. The exception is the voluntary reduction policy, where the base-level random forest model was used, and its recommendations were reduced by the percent reduction target.

It should be noted that our framework does not focus on the economic impacts of policies if simply applied to current market conditions. Instead, our approach reflects what would happen if agricultural researchers and farmers had to adapt to a new policy environment, meaning that data from field trials were used to optimize profitability after accounting for new costs under each policy scenario. These field-level calculations supported new N rate recommendations, which, as described next, were applied over the entire state to understand the impacts at scale across diverse biophysical production environments.

### 2.5. Stage 3: Evaluation Module

We first used the recommendation tools to make N rate predictions to the remaining 4,030 evaluation fields using the leave-one-year-out approach to evaluate each policy's economic impacts. The predictions were made in “ex-ante” conditions, at the maize stage of v5, with uncertainty about the growing conditions for the rest of the season. Farmers were assumed to apply the exact N rate recommended by the random forest tool, and we obtained the APSIM's output for that N rate and calculated the variables presented in Supplementary Table S3.

The profits were the profits obtained by farmers after paying any tax or fee to the government. The government collections were the funds collected by the government from those taxes or fees. The policy cost was the reduction in farmers' income after the government collections were returned and represented the deadweight loss due to the policy. The results at the field level were aggregated. This aggregation was calculated as follows (1) across years, giving each year the same weight, (2) for the region, giving each field the same weight, and (3) at the state level, also giving each field the same weight.

The N leaching in the simulations was measured during the 2-year period, including the maize year when N was applied and the following soybean year, to capture any residual effect of the N rate (Iqbal et al., 2018; Pasley et al., 2021; Mandrini et al., 2022). The environmental performance of the policies was evaluated by the reduction in N leaching relative to the base-level situation, as an absolute value (kg/ha) and a relative value (%).

Compensation was paid to farmers as a lump-sum per ha equal for all fields in the region. It had two components. The first component was a return of the government collections. For this, the total collections from the fields in each region were divided by the fields' total area and returned to farmers as an equal payment per ha and year. The second component consisted of a payment to cover the policy cost. These were “extra funds” that society pays farmers as a reimbursement for the loss in profits after the policy is implemented. It was also calculated at the region level and returned on a per-ha basis, meaning that all fields in a region received the same amount per ha and year. In the case of the voluntary reduction policy, since there were no government collections, the compensation included only the policy cost.

The main assumptions of this work are supported by considerations made during the optimization phase. The first assumption is that farmers are risk-neutral, profit-maximizer entities that apply the N recommendation tool's N rate. This assumption side-steps any behavioral considerations that affect farmers' decisions which have been studied in other works (Finger, 2012).

A second assumption is that the compensation does not offset the policy influence on farmers' N rate decisions. To warrant this, we set the compensation as a lump-sum per region, to ensure that it did not block the policies' desired effect. If the compensation was paid with a field-scale criteria, the same amount collected from a particular field would be returned to that same field. In that case, farmers will decide the N rate to use, knowing that any policy-related cost incurred will be recovered, and in consequence, will not reduce their N rates. The lump-sum mechanism avoids that and makes the return independent of the selected N rate for an individual farmer. We implemented policies with a regional criteria, but other spatial scales can also be considered.

A third assumption is that there is no land-use change with the policies, meaning that the rotation was maintained as soy/maize across different sub-levels of the policies. This is also warranted by the compensation mechanism, implementing it as a condition for planting maize. That means that farmers that planted maize will receive a lump sum, which will keep their income similar to the base-level income, and thus, the policy will not affect their land-use decisions.

A fourth assumption is that there are no administrative or transaction costs for monitoring the policies or transfers of funds between farmers and the government. These costs are currently unknown, and this allows for simplifying the calculations. Our aim was to evaluate policy effects driven by soil and climate variability, and if large differences are found for leaching or cost-effectiveness, then administrative and transaction costs could be included in future work.

A fifth assumption is that there is no other price response (for maize and N) outside the immediate price changes induced by the policies. For example, if N is too expensive and farmers reduce their N use to maintain profitability, maize production will decrease in the state, potentially increasing maize price. If maize price increases, farmers will re-calculate their EONR to higher rates, offsetting the initial desired effect of the policy. To avoid such effects, we excluded the levels of policies that lead to a decrease in yield at the region level by more than 5% of the base-level situation. More extensive policy modifications require other types of studies to predict how different supply chain actors would react and how that would affect grain stocks and prices globally.

## 3. Results

### 3.1. Policy Effects on Economic and Environmental Variables

**Figure 2:**
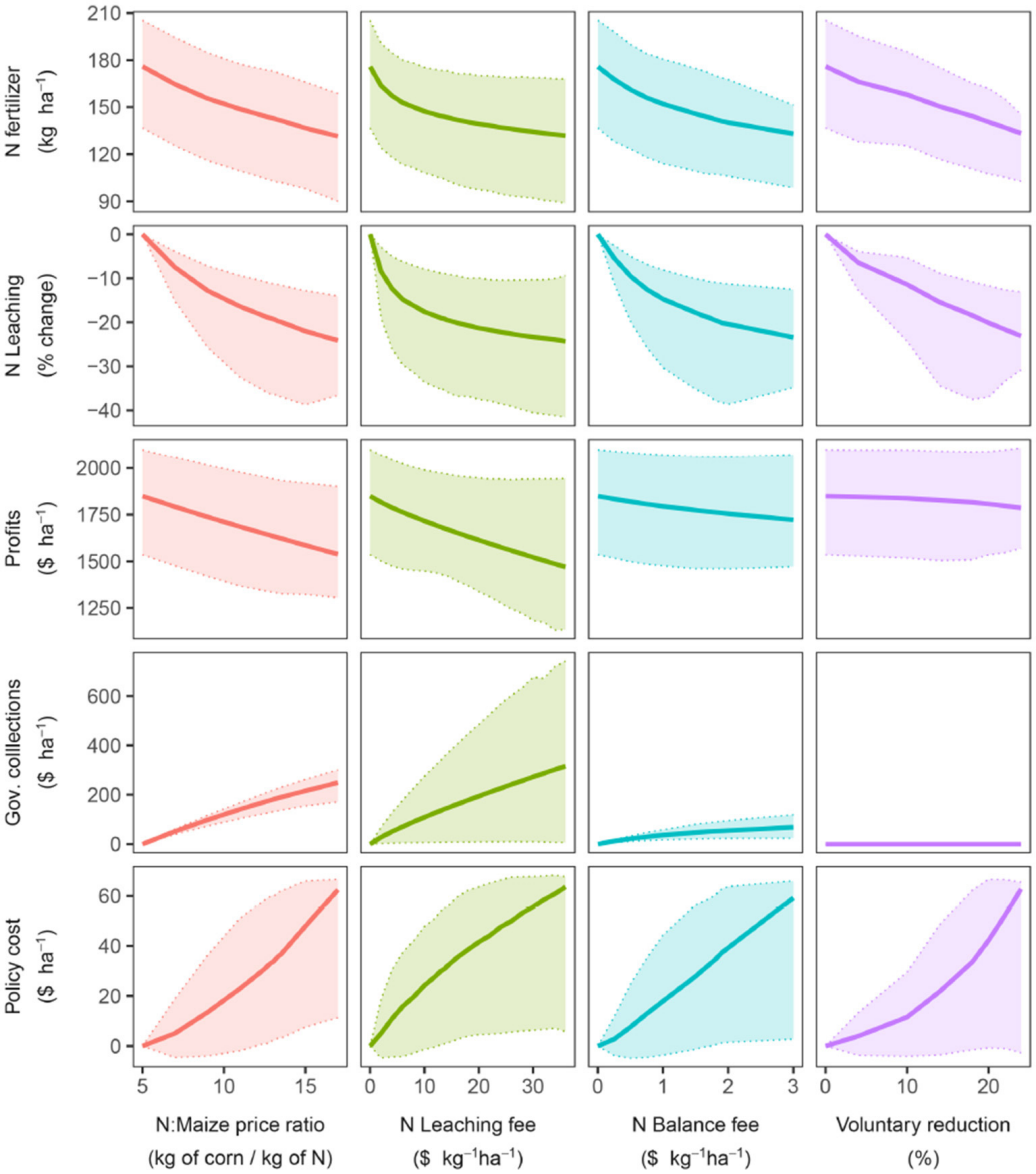
Trajectory of N fertilizer use, N leaching, Farm profits, government collections, and policy cost for the four policies. Solid line shows the mean. The shaded area indicates the 10 and 90% quantiles across fields (averaged across years).

Aggregated results at the state level indicate that the different policies work with a similar chain of effects (Figure 2). Initially, higher sub-levels of the policies caused a decrease in N fertilizer use. The reduction in N fertilizer translated into a decline in N leaching, yield, and farm profits. The government collections increased with higher sub-levels, except for the voluntary reduction since it does not involve collecting funds from farmers. The policy cost increased, indicating that the restriction on N fertilizer inputs induced by the policies caused a yield penalty that hurt farm profits to a greater extent than the funds collected by the government.

It is important to note that the fertilizer N rate in the ratio policy shows what in economics is known as the demand curve for N fertilizer. N fertilizer at the base-level situation had an own-price elasticity of 0.17, meaning that a 1% rise in price will decrease the quantity demanded by only 0.17%.

### 3.2. Cost-Efficiency of the Different Policies

**Figure 3:**
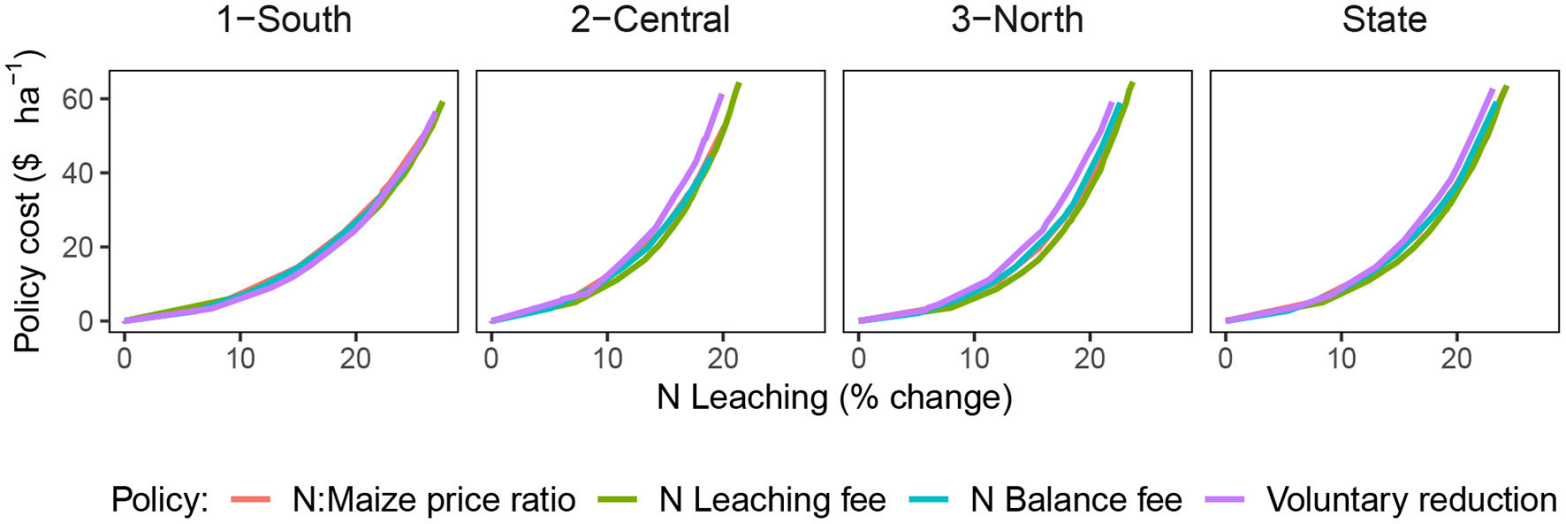
Efficiency of Policies for Reducing N-NO_3_ leaching relative to the base-level situation. Solid line shows the mean across all fields and years.

From a policy-maker's perspective, it is essential to find ways to achieve the desired environmental standard at the least cost. For that, we compared the cost of reducing N leaching for the different policy alternatives. Overall, the policy cost increased exponentially with higher N leaching reductions (Figure 3). Results suggest that a 10% state-level reduction can be obtained with an estimated cost of 8–10 US$/ha, and a 20% reduction with an estimated cost between 30–37 US$/ha. The different policies followed a similar trajectory, with a similar trade-off between economic and environmental goals.

### 3.3. Regional and Field-Level Effects at a 20% N Leaching Reduction

To further illustrate the effect of different policies, we selected the sub-levels of the policies that allow for a 20% state-wise N leaching reduction. After compensating farmers, policies reduced leaching by 20% while keeping farm income unaffected at the regional level (Table 1).

**Table 1:** Main indicators for the base-level situation and the four policies.

**Policy**		**Sub-** **level**	**Yield** **(*tonha*^−1^)**	**Leaching** **(*kgha*^−1^)**	***N* fertilizer** **(*kgha*^−1^)**	**Profits** **(*$ha*^−1^)**	**Gov. ** **collections** **(*$ha*^−1^)**	**Policy** **Cost ** **(*$kg*^−1^)**	**Abatement** **cost** **(*$kg*^−1^*ha*^−1^)**
*State*									
	Base-level	-	12.6	38.8	176	1849	-	-	-
	N:corn price ratio	12.9^(a)^	12.2	31.4	143	1637	179	33	4.5
	N leaching fee	13.2^(b)^	12.2	31.4	144	1682	138	30	4.1
	N balance fee	1.8^(c)^	12.2	31.2	142	1763	52	35	4.6
	Voluntary reduction	18.5^(d)^	12.2	31.4	143	1812	0	37	5
									
*1-South*									
	Base-level	-	12.1	35.9	193	1754	-	-	-
	N:corn price ratio	12.9^(a)^	11.7	27.9	159	1522	199	34	4.3
	N leaching fee	13.2^(b)^	11.7	27.9	160	1598	124	32	4
	N balance fee	1.8^(c)^	11.7	27.6	158	1658	60	36	4.4
	Voluntary reduction	18.5^(d)^	11.6	27.2	156	1712	0	42	4.8
									
*2-Central*									
	Base-level	-	12.5	37.0	176	1844	-	-	-
	N:corn price ratio	12.9^(a)^	12.2	30.5	144	1627	180	37	5.7
	N leaching fee	13.2^(b)^	12.2	30.7	146	1680	131	32	5
	N balance fee	1.8^(c)^	12.1	30.3	143	1755	49	39	5.8
	Voluntary reduction	18.5^(d)^	12.1	30.5	143	1801	0	43	6.6
									
*3-North*									
	Base-level	-	13.2	43.8	158	1954	-	-	-
	N:corn price ratio	12.9^(a)^	12.8	35.9	125	1769	156	29	3.7
	N leaching fee	13.2^(b)^	12.8	35.8	126	1770	158	26	3.3
	N balance fee	1.8^(c)^	12.8	36.0	126	1879	47	28	3.6
	Voluntary reduction	18.5^(d)^	12.8	36.7	128	1928	0	26	3.7

*Each policy is at the sub-level that allowed to obtain a state-wise reduction in leaching of 20%. Indicators' values are the average across all fields and years*.

(a) *kg corn/kg N*.

(b) *$ Nkg^-1^ ha^-1^*.

(c) *$ Nkg^-1^ ha^-1^*.

(d) %.

The table shows the sub-levels of the policies that achieve the 20% reduction (N:maize price ratio of 12.9 kg maize/kg N, N leaching fee of 13.2 US$ kg^-1^ ha^-1^, N balance fee of 1.8 US$ kg^-1^ ha^-1^, and a voluntary reduction of 18% from base-level N rate). Final N fertilizer use will be similar for different policies at these sub-levels, which is expected since the sub-levels were selected to achieve the same N leaching reduction. Additionally, final N fertilizer use varied across regions, given that the starting base-level N rate and N leaching are different.

The government collections varied across policies. These differences are driven by how the policy instrument is implemented. For example, the N leaching fee allows regulatory bodies to set an N leaching threshold, below which no fee is collected, reducing the government collections, while the N:maize price ratio modification has to be implemented over all N fertilizer sales, not allowing for such considerations. In our work, the size of the government collections had no practical importance since they were returned to farmers as part of the compensation.

Finally, the policy cost and abatement cost show the social cost of pollution control. When comparing policies, the N leaching fee had the lowest mean policy cost and abatement cost at the state level and in the individual regions. When comparing regions, the north had the lowest policy cost and abatement cost, and the central region had the highest.

Inside each region, the policies affected fields differently, modifying leaching and income more in some fields than in others. We calculated three types of field-level effects, using data at the field level averaged across all the years.

**Figure 4:**
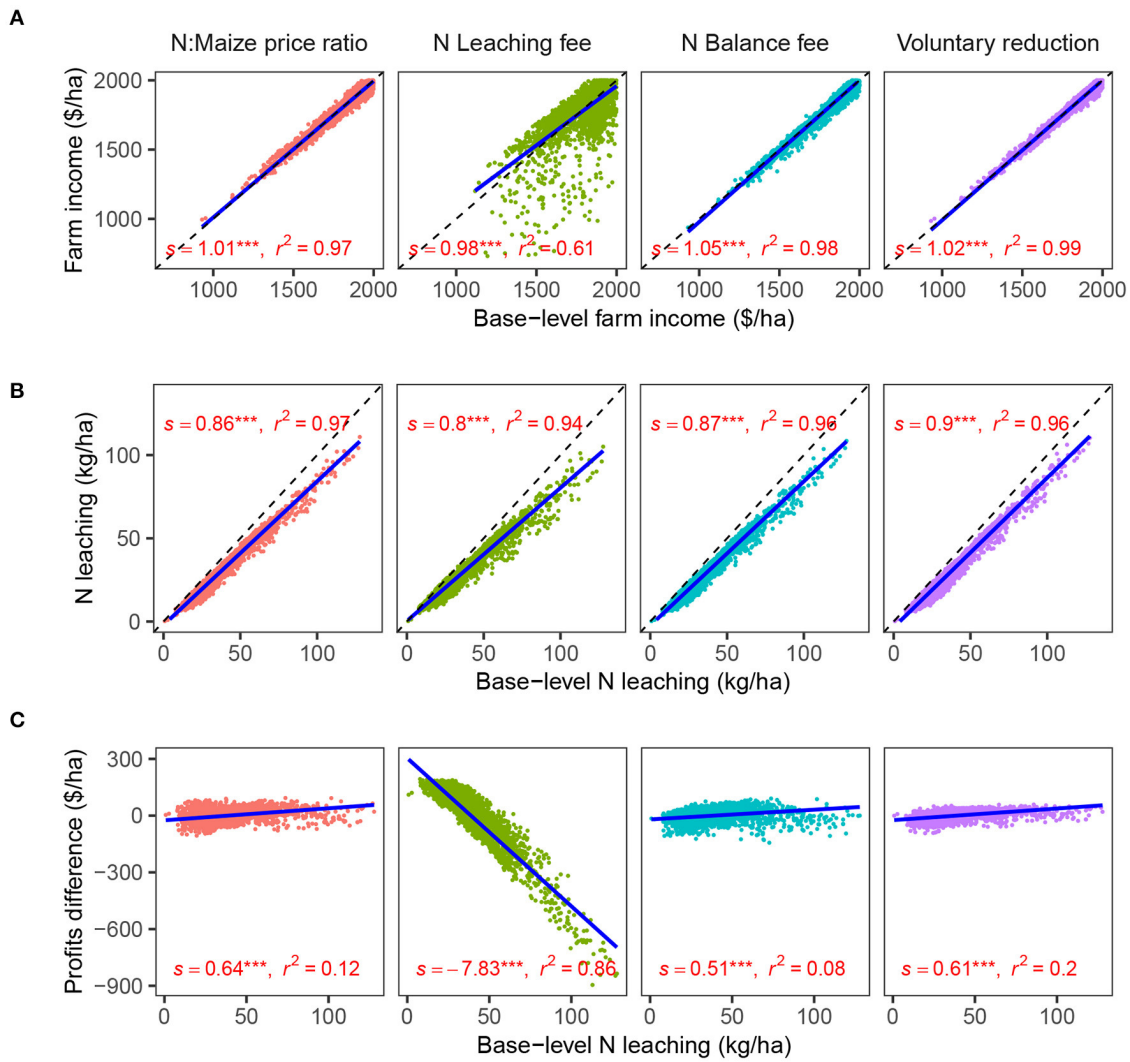
Field-level effects for the different policies compared to base-level indicators: **(A)** Farm income (farm profits + compensation) relative to the base-level income (income effect). **(B)** N leaching relative to the base-level N leaching (point source reduction effect). **(C)** Profits difference (farm profits after policy—farm profits before policy) relative to the base-level leaching (internalization effect). Observations are at the field level, averaged across years. Linear regressions were fitted independently to each set of data, and report the estimate of the slope (s), test of significance (ns = non-significant; *= *p* < 0.1, **= *p* < 0.05; ***= *p* < 0.01), and coefficient of determination (*r*^2^).

The “income effect” (Figure 4A) reflects changes in income (Equation 16, Supplementary Table S3) with respect to the base-level profits within each field. The price ratio modification, the N balance fee, and the reduction policy reduced N leaching and kept individual field-level income relatively unchanged. These three policies reallocated income from fields with high N rate needs to fields with low N rate needs (Supplementary Figure S4A), which explains the dispersion around the line. Nevertheless, balancing out the compensations by regions helped to contain this effect and reduce the overall impact. As explained below, the leaching fee policy showed a higher dispersion, with a reallocation of income from high leaching fields to low leaching fields.

The “point source reduction effect” (Figure 4B) reflects changes in N leaching relative to the base-level leaching within each field. It is desirable for a policy to reduce N leaching more in fields where N leaching is higher. This has environmental benefits since it will reduce hotspots where N concentration in water bodies can reach extreme values. While the four policies showed a good performance in this effect, the N leaching fee had the best performance (illustrated by the lower and significant slope of the regression line).

The “internalization effect” (Figure 4C) assessed how profits changed with respect to the base-level leaching. Ideally, a policy will decrease profits more in fields with high externality costs. Economically, internalizing these externalities allows firms to make decisions considering the cost to society, solving the market failure, and bringing production to socially optimal levels. Moreover, internalizing the externality will create market demand for more efficient N products, management practices, and crop rotations that would further reduce N leaching. The only policy that showed an internalization effect was the N leaching fee policy, and fields with higher leaching experienced higher income losses. Interestingly, the N balance fee did not show an internalization effect, and N balance did not well represent N leaching when the variation in N leaching in the data is due to soil and weather and not as much to N rates (more details in section 3.4).

### 3.4. N Balance as an Indicator of N Losses

**Figure 5:**
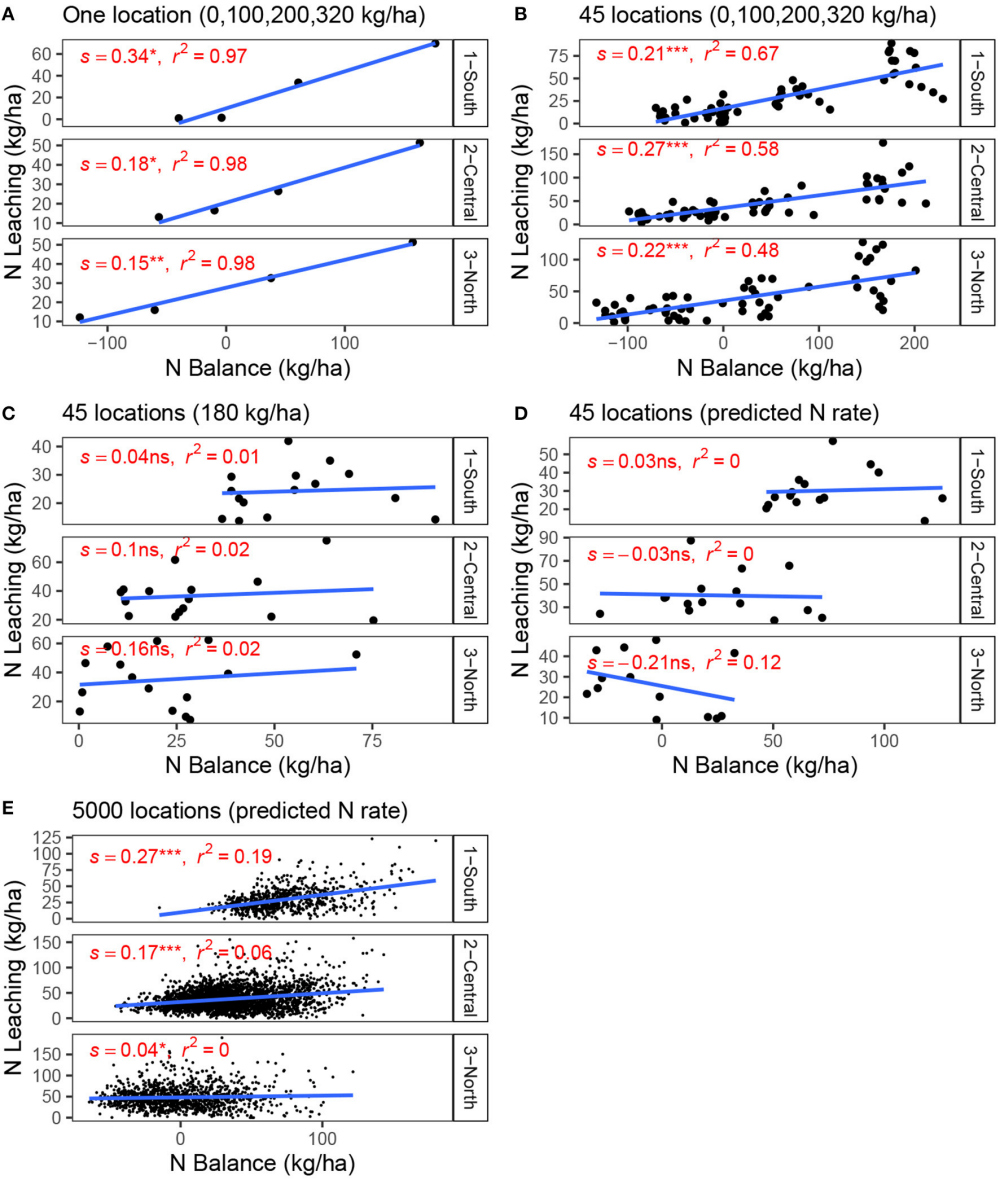
N balance vs. N leaching relationship with the increasing complexity of the dataset. Locations are a field x year combination randomly selected from the 128,100 available locations (4270 fields x 30 years). **(A)** One location with four fixed N rates (trial). **(B)** A total of 45 locations with four fixed N rates (trials). **(C)** Several locations using the same fixed N rate. **(D)** A total of 45 locations using the N rate predicted by the random forest tool at base-level situation. **(E)** A total of 5,000 locations using the N rate predicted by the random forest tool at base-level situation. Linear regressions were fitted independently to each set of data, and report the estimate of the slope (s), test of significance (ns = non-significant; *= *p* < 0.1, **= *p* < 0.05; ***= *p* < 0.01), and coefficient of determination (*r*^2^).

The ability of N balance to represent N leaching weakens with a comprehensive dataset where variation in N leaching is primarily due to soil and weather conditions and not as much due to variation in N rates across fields. Similar to previous work, our results suggest N balance correlates with N leaching when measured using trial information when multiple N rates are compared (Figures 5A,B). In this case, the N leaching variation is due to N rate variation, and N balance is a good indicator of N losses (McLellan et al., 2018; Eagle et al., 2020).

On the contrary, when the relationship is mapped using data with one N rate per field, our simulations suggest N leaching depends on complex soil processes that are not captured by the components of N balance. For example, prior research has attributed this to the asynchrony between soil mineralization and crop uptake (Martinez-Feria et al., 2018). To illustrate this, we show what happens with N balance if all farmers apply the same N rate (Figure 5C). In that case, the N balance is poorly associated with N leaching (non-significant slopes, and *r*^2^ < 0.02). As a result of similar N inputs, high-yielding fields have a lower N balance than low-yielding fields, and a policy that implements a fee on N balance will charge lower fees to those high-yielding areas to the detriment of the low-yielding areas. Nevertheless, there is no clear evidence that low-yielding fields have higher N leaching. Sometimes, the opposite happens and low-yielding fields tend to have lower mineralization (due to lower soil organic matter), and therefore a larger proportion of crop N demand is met by N fertilizer inputs, which means that an increase in N balance would correspond with lower N leaching losses. Similarly, high-yielding fields have soils with higher organic matter, decreasing the proportion of crop N demand met by N fertilizer inputs, and, despite a lower N balance, they have higher N leaching losses.

If instead of applying the same N rate across fields, farmers apply the N rate recommended by the random forest recommendation tool (which would be an improvement from many of the methodologies currently used), the N balance also does not accurately predict N leaching (Figures 5D,E). In this case, the optimal N rate recommended for a particular field is determined in part by crop, soil, and weather variables. These factors also affect the grain N removal component in Equation (2). Therefore, understanding the overall relationship between N balance and N losses is challenging (Sela et al., 2019) and could vary by region, depending on the interactions among those factors.

## 4. Discussion

### 4.1. Policy Implications on Agricultural and Environmental Outcomes

Balancing the profitability and environmental impact of agriculture is a pressing challenge. Our study provides actionable information for decision-makers about economic and environmental trade-offs for maize production, and it is particularly timely as the US Midwest enters a new era where there is growing interest in policies to address N pollution (Khanna et al., 2019; Kanter et al., 2020). The premise is that different policies can be used to achieve HTF goals. However, considering how the agricultural sector reacts to a policy and how that will translate into regional changes in economic and environmental performance, it is difficult to predict the net impacts of policies designed to induce lower N fertilizer use. Covering roughly 12 million ha of agricultural area and 30 years of simulations, our analysis provides important insights about the magnitude of trade-offs between crop production and N leaching losses under different policy scenarios. Mandrini et al. (2021) showed that dynamic recommendation tools reduce N leaching by 5% from the current recommendation system adopted by the extension services of seven Land Grant Universities in the Midwest (Maximum Return to Nitrogen (Sawyer et al., 2006). In the present article, we explored how policies could reduce N leaching even further. We focus on Illinois because it contributes to 20% of loads of N into the Mississippi river, with 86% of those loads generated by agriculture (Illinois-EPA, 2015).

When policies are implemented, they enable setting a sub-level that lowers farmers' N rate to meet a specific reduction in N leaching (Figure 2). The level at which a given policy allows for achieving a specific N use reduction depends on the price elasticity of N inputs. Our calculation agrees with previous studies that found N elasticity to be less than one (Roberts and Heady, 1982; Denbaly and Vroomen, 1993; Finger, 2012). This explains why policies need to produce large modifications in prices or fees to achieve changes in nitrogen use (Falconer and Hodge, 2000; Finger, 2012). For example, the price of N fertilizer needs to increase 2.6 times to reduce N demand by 19%, which would translate into a 20% reduction in N leaching (Table 1).

The N leaching fee achieved the lowest statewide policy cost, followed by the price ratio, the N balance fee, and the voluntary reduction (Figure 3). The reasons are in the optimization process during which the EONR of the trials is selected based on the condition of the policies which was used to train the random forest model (Figure 1). Incorporating N leaching directly with a fee makes the EONR lower in trials where higher N rates lead to higher N leaching (Figure 4B, Supplementary Figure S4C, Supplementary Section S5), achieving the highest efficiency in the reduction of N leaching. The price ratio policy follows an indirect way of reducing N leaching by making N fertilizer more expensive. This reduces the EONR in conditions where the marginal profits are not enough to cover the increased cost of N fertilizer. Such conditions are not necessarily the conditions that lead to higher N leaching, therefore the efficiency is lower. Similarly, the N balance indicator showed a lack of correlation with N leaching (Figure 5), which means that the EONR used for training the random forest model were lower in conditions that did not necessarily lead to higher N leaching. Finally, the voluntary reduction simply reduces N rates by a certain percentage, which is not necessarily the optimal way of reducing N rates across fields. Our optimization process symbolizes the decision-making process that thousands of farmers will perform when choosing their N rates given the new conditions of each policy.

At the field level, the N leaching fee showed additional benefits over other policies. First, it allows a better point source reduction effect (Figure 4B). Second, it showed a better internalization effect (Figure 4C). Nevertheless, that better internalization effect causes a reallocation of income from high leaching to low leaching fields (Figure 4A). This needs to be addressed by policy-makers since it would increase the political cost of charging a fee on N leaching, especially because fields that have high N leaching will see their income decrease.

The cost-efficiency analysis suggests that an appropriate target is to use these policies to reduce leaching by 20% since the cost of the reduction raises sharply afterward. The 20% reduction comes with a policy cost of 34 US$/ha (average for the four policies). If these estimates are scaled up to the yearly area of maize production in the state (4.4 million ha NASS, 2019), this will represent a policy cost of 147 million US$/year, reducing 32.6 million kg/year of N loads.

Welfare can be understood by the sum of after policy farmers' income (profits + compensation) and the environmental damage caused by nitrate losses (by assuming that tax, fees, and compensations are monetary transfers between the farmers and the rest of society, we can suppress them from the welfare computation). Considering that the environmental cost from groundwater contamination is 16.1 US$ per kg N (Sobota et al., 2015; Jin et al., 2019) (including undesirable odor and taste, nitrate contamination, increased colon cancer risk, and increased eutrophication), our simulated dataset suggests that reducing N fertilizer use will reduce externalities by an estimated 524 million US$/year. This represents an increase in the welfare of 377 million US$/year and a return on investment of 260%, showing how beneficial it is to reduce N loading upfront rather than handling the externalities associated with environmental pollution and human health damages.

Moreover, the policy cost of 147 million US$/year to be compensated by the government can be compared to current spending on the state of Illinois by conservation programs that target farmer N management during the year 2020, like the Conservation Reserve Program (CRP), with a cost of 167 million US$/year (Farm Service Agency, USDA, 2020), Environmental Quality Incentives Program (EQIP) with a cost of 21.9 million US$/year (Natural Resources Conservation Service (NRCS), 2020b), and Conservation Stewardship Program (CSP) with a cost of 87.2 million US$/year (Natural Resources Conservation Service (NRCS), 2020a). It can also be compared with crop insurance subsidies, whose payment represents 373 million US$/year (Risk Management Agency, USDA, 2020). It has been suggested that there is substantial potential to redirect farm support toward environmental goals (Searchinger et al., 2020). In this context, using governmental funding to compensate the economic losses of farmers due to lower N inputs at the farm level should be further explored in terms of its political and economic feasibility to become a promising solution.

The scenarios evaluated in this study illustrate how the proposed policies might contribute to an appealing new set of options that could be incorporated into voluntary efforts such as the Illinois NLRS, due to their low cost and high potential for total N leaching reductions. The NLRS identified several strategies that can be used to meet statewide N loss reduction goals and compared their cost per kg of N removed and the potential nitrate reduction that each strategy would allow if implemented (reproduced in Supplementary Table S4). Relative to these NLRS strategies, at the level that allows a 20% reduction (Table 1), our policies would be the second in terms of potential nitrate reduction (32.6 million kg of N reduced) and the fourth in cost (4.1–5 US$/kg removed). In contrast to practices that require important changes in the production system, N rate reduction policies are highly compatible with the other practices (in-field and edge-of-field practices), making it possible to combine them, adding their potential to reduce N leaching overall (Christianson et al., 2018).

There are essential differences among the regions, driven by increases in soil organic matter and better-growing conditions when moving south to north (Supplementary Figure S3). This spatial heterogeneity reinforces the need for local adjustments of policy thresholds and calculating the compensations to the farmers by region. If we did not calculate the compensation by region, perverse effects resulting from policy implementation would benefit farmers in some regions to the detriment of other regions. Similar results against uniform implementations of policies were obtained in France (Jayet and Petsakos, 2013).

It is estimated that in the US, US$1.9 trillion has been invested in improving water quality since 1960, exceeding the cost of most other US environmental initiatives. However, this has resulted in uncertain and relatively low benefits according to 20 recent assessments (Keiser et al., 2019). Most of these more effective investments have been aligned with the “pay the polluter” approach, through which financial and technical assistance to farmers are used to encourage and support voluntary adoption of pollution controls. Our voluntary reduction policy falls into this category.

Alternatively, the “polluter pays principle” is proposed as a paradigm change needed to start achieving the desired environmental results in agriculture (Shortle et al., 2012). The price ratio, the N leaching fee, and the N balance fee policies proposed have features aligned with the polluter pays principle. Even though we included compensation to keep income levels similar, in these policies, farmers face higher costs if they pollute, and the compensation is a lump-sum independent of their pollution level. Polluter pays policies have several advantages. First, they are supply-driven, and farmers in their self-interest respond to the policy's new economic conditions instead of programs that depend on voluntary adoption of different practices. Second, they do not create incentives that could inadvertently work in opposition to the policy's goal, as could happen with subsidies that in some situations encourage farmers to increase production or maintain production in environmentally sensitive locations. Finally, the mechanism that induces farmers to lower their pollution (tax or fees) generates revenue, making their implementation independent of public expenditures, often limited by budget constraints.

### 4.2. Strength and Weaknesses of Each Policy

In our results, all policies reduced N leaching, with the N leaching fee showing the highest statewide cost efficiency, a better point source reduction effect, and a stronger internalization effect. Despite these benefits, any decision of which policy is the most promising to explore should also consider the political cost, administrative cost, expected adoption rate, and the simplicity of implementation.

The strength of the price ratio modification is its simplicity in implementation and low monitoring cost, since farmers will pay the tax when purchasing fertilizer without measuring any other variables. Another strength is its high response rate since farmers will respond to new prices to maximize their profits. The limitations are the negative perceptions around raising taxes on agricultural production inputs in the US, especially considering that the inelastic demand requires the N price to increase 2.6 times for a 20% N leaching reduction (Table 1).

The leaching fee policy's main strength is being performance-based, aligning farmers directly with the problem to solve, which allows them to find the most efficient way of reducing it. It will also have a high response rate since the fee will charge all farmers above a certain leaching threshold. The disadvantages include finding ways of measuring N leaching at a big scale. Monitoring non-point source pollution at the field level has not been possible previously, largely due to technical constraints that have limited this option for policy development. However, new technologies, like computer modeling, are being developed to track N losses in each field (Shortle et al., 2012).

The N balance fee provides an easy-to-calculate indicator that can be easily understood. The main problem with this policy is that N balance was not a particularly strong predictor of N losses in our simulations, especially with comprehensive field and weather data, coupled with high variability in soil characteristics and low variability in N rates (Figure 5). Other work has shown that N losses are related to soil organic matter mineralization and poor soil N retention as much as inefficient N fertilizer use (Martinez-Feria et al., 2018). The N balance equation does not consider the contributions of those soil N cycling processes, which are a significant part of the final N leaching.

Therefore, researchers and policymakers should consider that N balance fees could help lower N rates from farmers who apply excessive N fertilizer in their fields, without any yield advantage than other fields (Tenorio et al., 2020). However, in the scenario for this study where farmers are using a recommendation tool to predict the EONR and applying N at side-dress, the N balance is at risk of not showing farmers' environmental impacts. Creating multiple regions that cluster fields with similar soils, climate, and cropping systems can help control this effect, and the N leaching variation will depend more on the N rate selection captured by the N balance. Another alternative is to develop N use efficiency indicators that account for soil organic matter mineralization and soil N retention to improve the prediction accuracy of N leaching losses. This aspect requires awareness and further research.

The voluntary reduction policy's strength is its low political cost since it is designed as a voluntary approach, which farmers usually perceive as better. The main weakness is the likelihood of low adoption. Research into farmer decision-making shows that several barriers exist to reducing N rates, mainly because N fertilizer is perceived as a risk-reducing factor that ensures high yields (SriRamaratnam et al., 1987; Sheriff, 2005; Reimer et al., 2018). Therefore, those who attempt to persuade producers to reduce N applications face significant challenges (Stuart et al., 2014).

An important consideration for all policies is the likelihood of generating positive spillover effects or synergies in the system that could increase the environmental benefits. As an analogy to another pollutant, when oil price increases, it discourages fuel use in the short-term and increases the demand for more fuel-efficient or electric vehicles and public transportation in the long term (Fischer et al., 2007; Parry et al., 2007). Similarly, the policies explored in our work could encourage more efficient N management besides the reduction in N fertilizer consumption explored here. Such synergies are possible in the ratio, leaching fee, and balance fee policies. Those policies can raise the adoption of practices and technologies that increase N use efficiency, i.e., enhanced fertilizers, split applications, cover crops, and others. For example, Sela et al. (2019) evaluated how N balance levels changed by improved timing and formulation of fertilizer applications. A larger market for efficient technologies would in turn create incentives for the private and public sectors to invest in research and launch products that help save N fertilizer inputs. It will also increase incentives for breeding organizations to focus on more N-efficient genetics. One of these practices, cover crops, has also been identified as one of the most effective “nature-based” carbon mitigation approaches (Fargione et al., 2018).

The N leaching fee is likely to generate more effective spillovers directly aligned with the problem. The price ratio and N balance policies focus on fertilizer use and induce spillovers on practices that increase N use efficiency. The N leaching fee will create demand for any practice that reduces N leaching by any means, not only by increasing N use efficiency. For example, suppose cover crops in some areas reduce N leaching but have no effect on the system's N fertilizer input need. In that case, the price ratio policy or the N balance fee will not increase their adoption, but the N leaching fee will. Contrary to those three policies, the reduction policy is unlikely to create spillovers besides the N rate reductions. The reason is that it only accounts for farmers reducing their N rate voluntarily, and it does not create additional incentives for farmers to adopt other technologies that reduce N leaching.

### 4.3. Limitations of This Work

Among the limitations of our methodology, all our data were generated with process-based crop modeling combined with publicly available soil and weather information. Crop models are a useful means of representing complex plant-soil interactions based on current knowledge, yet there are inherent limitations to accuracy on an absolute basis. Accordingly, simulation results are helpful for scenario analysis but must be interpreted with caution. We have attempted to counter such limitations by calibrating and validating the data with actual field trials across many locations determining EONR. Also, we assumed that any bias of the model would be constant across soils and policies, and relative comparisons will remain accurate (Baum et al., 2020).

Additional limitations are related to the assumptions stated in the methodology section. Those assumptions may not necessarily hold. For example, the assumption that farmers are risk-neutral, profit-maximizer entities side-steps behavioral effects that a policy can cause: farmers sometimes apply N rates higher than the profit-maximizing N rates (Sellars et al., 2020), or farmers paying a high leaching fee or N balance fee one year may reduce their rates too much the following year.

Overall, our framework allows us to explore the impact of different policies on nitrogen-load reductions and derive the best theoretical policy. Therefore, simplifications in the agricultural system's representation were necessary to constrain simulation complexity and focus on adaptation effects. Our results must be interpreted within the context of the mentioned limitations and considered insights or relative values and not exact values.

## 5. Conclusion

Our regional-scale simulation results provided important insights for policymakers to make decisions that guide production toward a more eco-efficient resource use. Based on simulations for 4270 randomly selected fields in Illinois assessing the effects of four potential policies for reducing N rates, we concluded that: (i) policies allowed for a substantial reduction of N leaching with limited effects on crop productivity, up to 20 % with a cost of 30–37 US$/ha, but then the cost increased sharply, (ii) all policies showed a smoothing effect, reducing leaching more in areas with high leaching (hotspots). The N leaching fee was the only policy with an internalization effect, affecting more high N leaching fields. This is desirable from an environmental stand but will increase the political cost of the policy, (iii) the policies offer a new set of alternatives that can be incorporated into the current NLRS in the state, positioning them as the second in the total potential of reduction. Moreover, they can be combined with other solutions to increase the reductions even further, and (iv) policymakers should evaluate them in terms of political cost, administrative cost, the expected adoption rate, the simplicity of the implementation, and the spillover effects.

There are limitations to our crop modeling framework that suggest that our findings should be interpreted with caution. However, the conclusions provide insights on the relative advantages and disadvantages of different approaches for limiting N inputs and leaching losses at the field level, that could guide future policy discussions toward decreasing the environmental impact of N use. This will be an essential step in the path to the increase of the eco-efficiency of US agriculture.

## Data Availability Statement

Publicly available datasets were analyzed in this study. The article explaining the simulations can be found at: https://www.sciencedirect.com/science/article/pii/S2352340921010283. The data can be found at: https://data.mendeley.com/datasets/xs5nbm4w55/.

## Author Contributions

GM: conceptualization, data curation, formal analysis, investigation, methodology, software, validation, visualization, writing—original draft, and writing—review and editing. NM: methodology, resources, funding acquisition, and supervision. DK: methodology and writing—review editing. SA: methodology, validation, and writing—review editing. CP: conceptualization, methodology, validation, and writing—review and editing. All authors contributed to the article and approved the submitted version.

## Funding

This study was conducted thanks to the support of NIFA Hatch/Multistate Hatch Grant, Enhancing nitrogen utilization in corn-based cropping systems to increase yield, improve profitability, and minimize environmental impacts, ILLU-802-965.

## Conflict of Interest

The authors declare that the research was conducted in the absence of any commercial or financial relationships that could be construed as a potential conflict of interest.

## Publisher's Note

All claims expressed in this article are solely those of the authors and do not necessarily represent those of their affiliated organizations, or those of the publisher, the editors and the reviewers. Any product that may be evaluated in this article, or claim that may be made by its manufacturer, is not guaranteed or endorsed by the publisher.
