# Corrigendum: Exploring trade-offs between profit, yield, and the environmental footprint of potential nitrogen fertilizer regulations in the US Midwest

**DOI:** 10.3389/fpls.2022.1100580

**Published:** 2023-01-10

**Authors:** German Mandrini, Cameron Mark Pittelkow, Sotirios Archontoulis, David Kanter, Nicolas F. Martin

**Affiliations:** ^1^ Department of Crop Sciences, University of Illinois at Urbana-Champaign, Champaign, IL, United States; ^2^ Department of Plant Sciences, University of California, Davis, Davis, CA, United States; ^3^ Department of Agronomy, Iowa State University, Ames, IA, United States; ^4^ Department of Environmental Studies, New York University, New York, NY, United States

**Keywords:** environmental policy, bio-economic modeling, externalities, nitrogen pollution, nitrogen use efficiency

In the original article, there was an error in the units for the price ratio policy. They were inverted, being kg corn/kg N, instead of kg N/kg corn. The error was repeated throughout the article in equations, text, and figures. We are correcting the mistake, but it did not affect the conclusions.

The original footnote text was:

“Each policy is at the sub-level that allowed to obtain a state-wise reduction in leaching of 20%. Indicators’ values are the average across all fields and years.

(a) kg N/kg corn. (b) $ Nkg^-1^ ha^-1^. (c) $ N kg^-1^ ha^-1^. (d) %.”

The corrected footnote text appears below.

“Each policy is at the sub-level that allowed to obtain a state-wise reduction in leaching of 20%. Indicators’ values are the average across all fields and years.

(a) kg corn/kg N. (b) $ Nkg^-1^ ha^-1^. (c) $ N kg^-1^ ha^-1^. (d) %.”

There was also an error in **Figure 2** as published. The units for the price ratio policy are inverted.

**Figure 2:**
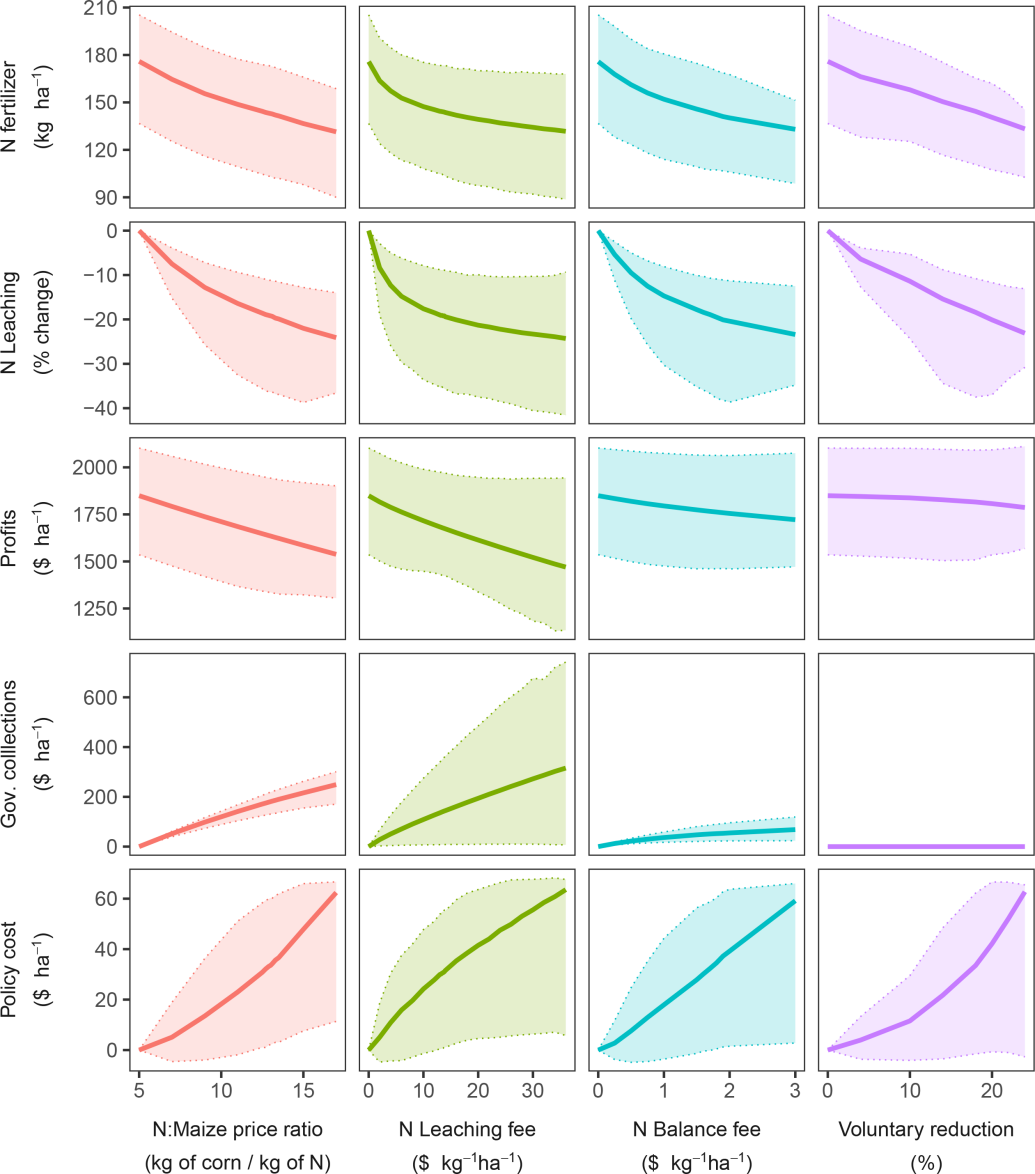
Trajectory of N fertilizer use, N leaching, Farm profits, government collections, and policy cost for the four policies. Solid line shows the mean. The shaded area indicates the 10 and 90% quantiles across fields (averaged across years).

These units have also been corrected in the following sections: **Methods**, Stage 2: Optimization Module, Policy Scenarios, N:maize price ratio modification, equation 1, **Methods**, Stage 2: Optimization Module, Policy Scenarios, N:maize price ratio modification, paragraph 3, **Results**, Regional and Field-Level Effects at a 20% N Leaching Reduction, paragraph 2.

The units previously stated:

“kg N/kg maize”

The corrected units appear below:

“kg maize/kg N”

There were formatting errors in the supplementary material. The supplementary material file in the original article has been updated. The authors apologize for these errors and state that this does not change the scientific conclusions of the article in any way. The original article has been updated.

