# Supplementary material for: Exploring Trade-Offs Between Profit, Yield, and the Environmental Footprint of Potential Nitrogen Fertilizer Regulations in the US Midwest
Source: Front Plant Sci. 2022 Apr 15;13:852116. doi: 10.3389/fpls.2022.852116 (PMC9051523; doi:10.3389/fpls.2022.852116)
Supplement: Supplementary file 1 [file Data_Sheet_1.pdf]

Supplemental Materials

S1 Database description

Table S1: Database characterization. Each row represented a simulation for one field in one year with one N rate, with a total of 4,227,300 rows (4270 field x 30 years x 33 N rates)

Variable	Description	Units	Use
field_id	field identificator number (1 to 4270)	-	-
year	year of the simulation (1989-2018)	-	-
type	trial field or evaluation field	-	-
N rate	Nitrogen added as fertilizer in v5	kg/ha	treatment
Yield	Yield of the corn in with 15% Moisture	kg/ha	response
Profits	Profits obtained by farmers (equation 3, 4 or 5)	$/ha	response
Leaching	Total 2-years N leaching during corn and soybean.From April 1st year (x) to March 31st year (x+2)	N kg/ha	response
Yld_lt_avg	Mean yield at EONR (for the other 29 years)	kg/ha	predictor
rain_1	Total precipitation during first period (1 Jan. to planting)	mm	predictor
rain_2	Total precipitation during second period (planting to v5)	mm	predictor
t_mean_1	Average air temperature during first period (1 Jan. to planting)	°C	predictor
t_mean_2	Average air temperature during second period (planting to v5)	°C	predictor
rad_1	Average solar radiation during first period (1 Jan. to planting)	$MJ/m^2/day$	predictor
rad_2	Average solar radiation during second period (planting to v5)	$MJ/m^2/day$	predictor
lai_v5	Leaf Area Index at v5	m^2/m^2	predictor
surface_wt_v5	Surface residue weight at v5	kg/ha	predictor
n_0_60cm_v5	Soil N (NO3 and NH4) from 0 to 60 cm at v5	kg/ha	predictor
esw_pct_v5	Extractable soil water (ESW) at v5	%	predictor
WHC	Water holding capacity	mm	predictor
SOM	Soil Organic Matter at v5 (0-20 cm)	%	predictor
sand	Sand content (0-20 cm)	%	predictor
clay	Clay content (0-20 cm)	%	predictor

S2 Historic price ratio

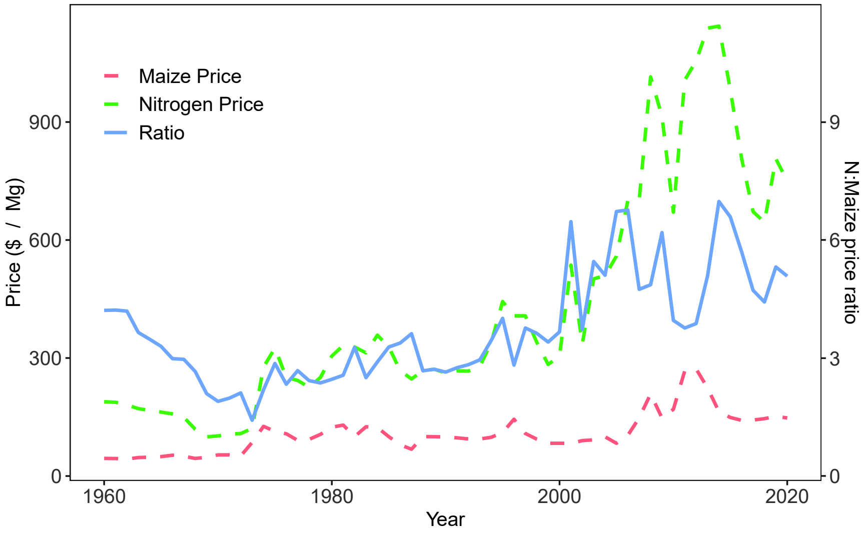

Figure S1: Historical N:maize price ratio (kg maize / kg N) evolution in the US from 1960 to 2020 (nominal values). Source: National Agricultural Statistics Service

S3 Optimization algorithm steps

Table S2: Description of the optimization process for the random forest recommendation tool, across the different policies. In all cases, the model was trained with the trial data from stage 1.

Policy	Algorithm steps
Price ratio	1. Profits were calculated, using the corresponding price of N with eq. 3 for each N rate on each response curve<br>2. The N rate that maximized profits for each response curve was selected and used as the training set<br>3. The random forest model was trained with the training set
Leaching fee	1. Profits were calculated, subtracting the corresponding fee on N leaching, using eq. 4 for each N rate on each response curve<br>2. The N rate with the highest profit on each response curve was selected and used as the training set<br>3. The random forest model was trained with the training set
Balance fee	1. Profits were calculated, subtracting the corresponding fee on N Balance, using eq. 5 for each N rate on each response curve<br>2. The N rate with the highest profit on each response curve was selected and used as the training set<br>3. The random forest model was trained with the training set
Voluntary reduction	1. The previous random forest model with a price ratio of 5 was used to obtain base-level predictions<br>2. Base-level predictions were reduced by the reduction target (%)

S4 Equations

Table S3: Equations used to obtain economic indicators in stage 2 optimization (i.e. for each N rate used in the trial) and for the stage 3 evaluation (i.e. for each N rate recommended by a model).

Stage used	Equation	Description	Eq
2	$P_{prcfzN} = Pc\, Y_{rcfN} - Pn_p N$	Profits obtained by farmers in the ratio policy	3
2	$P_{prcfzN} = Pc\, Y_{rcfN} - Pn_p N – (L_{rcfN} – Lt_r) Pf_p$	Profits obtained by farmers in the leaching policy	4
2	$P_{prcfzN} = Pc\, Y_{rcfN} - Pn_p N – (BrcfN – Bt_r) Pf_p$	Profits obtained by farmers in the N Balance policy	5
3	P_{prcfz} = Pc Y_{prcfz} - Pn_p N_{prcfz}	Profits obtained by farmers in the ratio policy	6
3	P_{prcfz} = Pc Y_{prcfz} - $Pn_{p=0}$ N_{prcfz} - $(L_{prcfz} - Lt_r)$ Pf_p	Profits obtained by farmers in the leaching policy	7
3	P_{prcfz} = Pc Y_{prcfz} - $Pn_{p=0}$ N_{prcfz} - $(B_{prcfz} - Bt_r)$ Pf_p	Profits obtained by farmers in the N Balance policy	8
3	P_{prcfz} = Pc Y_{prcfz} - $Pn_{p=0}$ N_{prcfz}	Profits obtained by farmers in the reduction policy	9
3	$GC_{prcfz} = N_{prcfz}$ (Pn_p - $Pn_{p=0}$)	Government collections in the ratio policy	10
3	GC_{prcfz} = (L_{prcfz} - Lt_r) Pf_p	Government collections in the leaching fee policy	11
3	GC_{prcfz} = (B_{prcfz} - Bt_r) Pf_p	Government collections in the N balance fee policy	12
3	$Pol\_cost_{prcfz} = P_{p=0,rcfz}$ - P_{prcfz} - G_{prcfz}	Deadweight loss due to the policy	13
3	$Abat\_cost_{prcfz} = \frac{PolicyCost_{prcfz}}{L_{p=0,rcfz} - L_{prcfz}}$	Abatement cost per kg of N leaching reduced	14
3	$Compensation_{pr...} = G_{pr...} + PolicyCost_{pr...}$	Compensation returned to farmers	15
3	$Income_{prcfz} = Compensation_{pr...} + P_{prcfz}$	Income obtained by farmers	16

Note: Y is the yield of maize (kg/ha), N is the N rate (kg/ha), L is the total N leaching (kg/ha), Lt is the leaching threshold (kg/ha), B is the N balance (kg/ha), Bt is the N balance threshold (kg/ha), G are the government collections ($/ha), Pc is the price of maize ($/ha), Pn is the price of Nitrogen fertilizer ($/kg). Pf is the price of the fee on N leaching or N balance in $ N kg $^{-1}$ ha^{-1} , defined by the sub-level of the policy. Subscript r is the rth region, c is the cth cell, f is the fth field, z is the zth weather-year, p is the pth policy level. Subscript p=0 means that the variable adopted the value of the base-level situation (current price ratio of 5, no tax, no fee). A dot replacing a subscript means the average across all the observations for that subscript.

S5 Variable importance at 20% reduction level

The importance of the different predictor variables for the random forest model was measured using the varImpPlot function in the R "randomForest" package. The varImpPlot function calculates the decrease in node impurities after splitting on the variable, averaged over all trees. For regression, the node impurity is measured by the residual sum of squares. Important variables are identified as those that reduce the residual sum of squares in the splits on which they were used (the difference between the residual sum of squares before and after the split).

The variable importance plot shows how the ranking of importance of the different variables changed with the policies (Figure S2). The most notable change is that the soil water content at the crop stage of v5 (which is usually high in this region, since soils receive rain and snow during the early spring) became important in the N leaching policy. Higher leaching is expected in conditions with higher soil water content, and this exemplifies how charging the pollutant directly makes the model account for variables that could help to recognize conditions where N leaching can be higher.

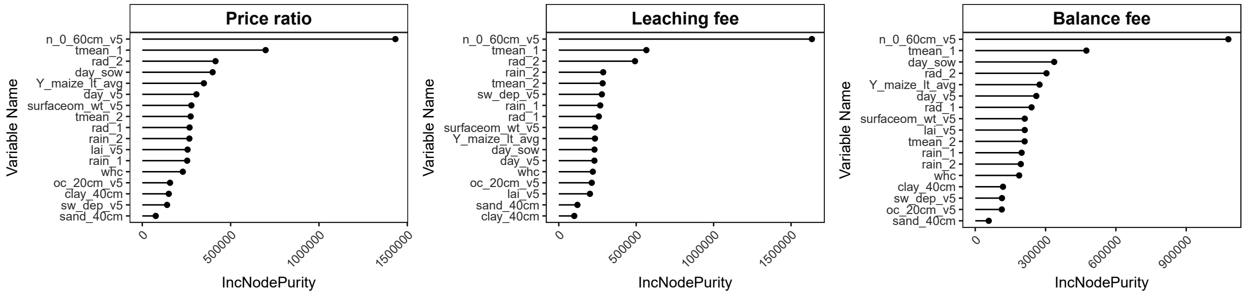

Figure S2: Variable importance plot, as measured by the node purity score (see text for details). One example for each policy, at the 20% reduction sub-level

S6 Probability density functions

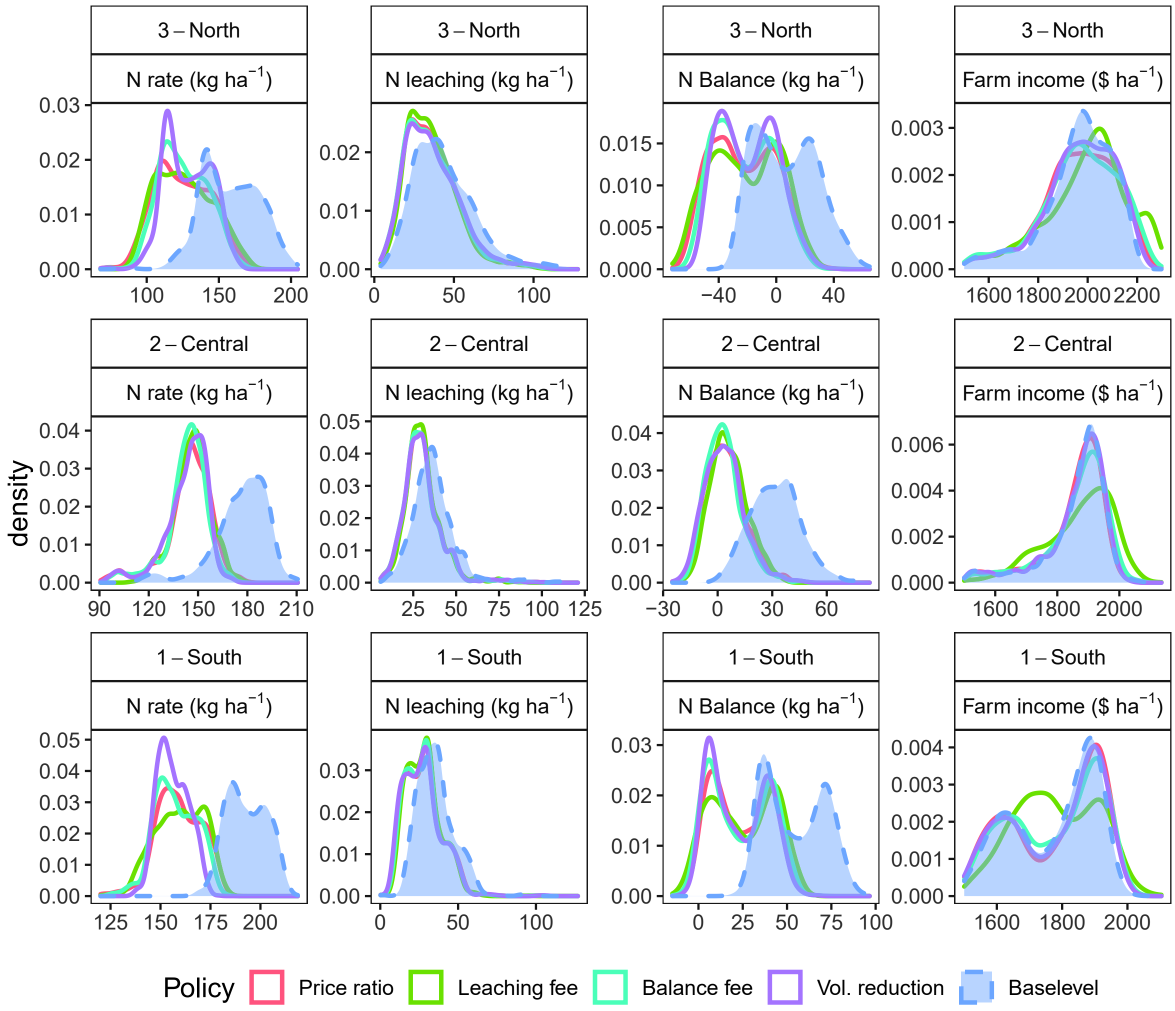

Figure S3: Density plots for key crop system processes, grouped by regions. Farm income = profits + compensation. Density plots show the distribution of a continuous variable, standardized so the sum of the area under the curve equals 1. Observations are at the field level, averaged across 30 weather years

S7 NLRS practices

Table S4: Comparison of practices incorporated in the Illinois NLRS and our policies. Created using Table 3.11.from Illinois-EPA 2015, adding our policies targeting 20% reduction in N-NO_3 losses (row in green)

	Cost ($/kg removed)	Nitrate-N reduced (million kg)
Reducing N rate from background to MRTN on 10 percent of acres	-9.4	1.0
Split application of 40 percent fall, 10 percent pre-plant, and 50 percent side dress	0.0	11.8
Buffers on all applicable crop land (reduction only for water that interacts with active area)	3.6	16.3
Our policies (20% sublevel)	4.1-5	32.6
Bioreactors on 50 percent of tiledrained land	4.9	15.9
Nitrification inhibitor with all fall-applied fertilizer on tile-drained corn acres	5.1	2.0
Spring-only application on tiledrained corn acres	7.0	11.8
Perennial/energy crops on 10 percent of tile-drained land	7.0	11.3
Cover crops on all corn/soybean tile-drained acres	7.1	38.1
Point source reduction to 10 mg/L	7.3	6.4
Wetlands on 35 percent of tiledrained land	8.9	22.2
Split application of 50 percent fall and 50 percent spring on tiledrained corn acres	13.7	5.9
Perennial/energy crops equal to pasture/hay acreage from 1987	20.6	4.5
Cover crops on all corn/soybean non-tiled acres	24.3	15.0

S8 More field effects

In section 3.3, we saw that the policies were capable of keeping profits similar to the base level, except for the N leaching fee policy that transfers funds from high leaching areas to low leaching areas (Figures 4a and 4c). We explored other possible transfers of funds that the policies could generate. First, policies that target the N rate directly or indirectly transfer funds from high N rate fields to low N rate fields (Figure S4a). This effect is noticeable in the price ratio policy ($r^2 = 0.247$) and in the N balance fee policy ($r^2 = 0.262$). The implication is that the policies will benefit fields with soil and weather conditions that lower their N rate to the detriment of fields requiring a higher N rate. This is not necessarily desirable since higher N rate needs do not always correlate with higher N leaching (Figure 5e), as N leaching depends on other soil and weather characteristics.

The second possible fund transfer we explored is from fields with a yielding capacity. This will be expected if a policy affects fields with different yields in a different way. For example, if two fields use the same N rate, but one field has a higher yield than the other, the N balance will be lower in the high-yielding field, possibly transferring funds from low-yielding fields to high-yielding fields. None of the policies showed a significant degree of this effect (Figure S4b), possibly because the higher yield was correlated with a higher N rate in some areas, which increases N balance, offsetting the effect.

Another field effect we analyzed is the change in N fertilizer recommendation with respect to N leaching (Figure S4c). It is desired that a policy will reduce N fertilizer more in areas

with high N leaching. The only policy to show this effect was the N leaching fee ($r^2 = 0.36$), showing how a first rank policy targeting the problem directly allows for more efficient reductions than second rank policies that target one of the causes of the problem.

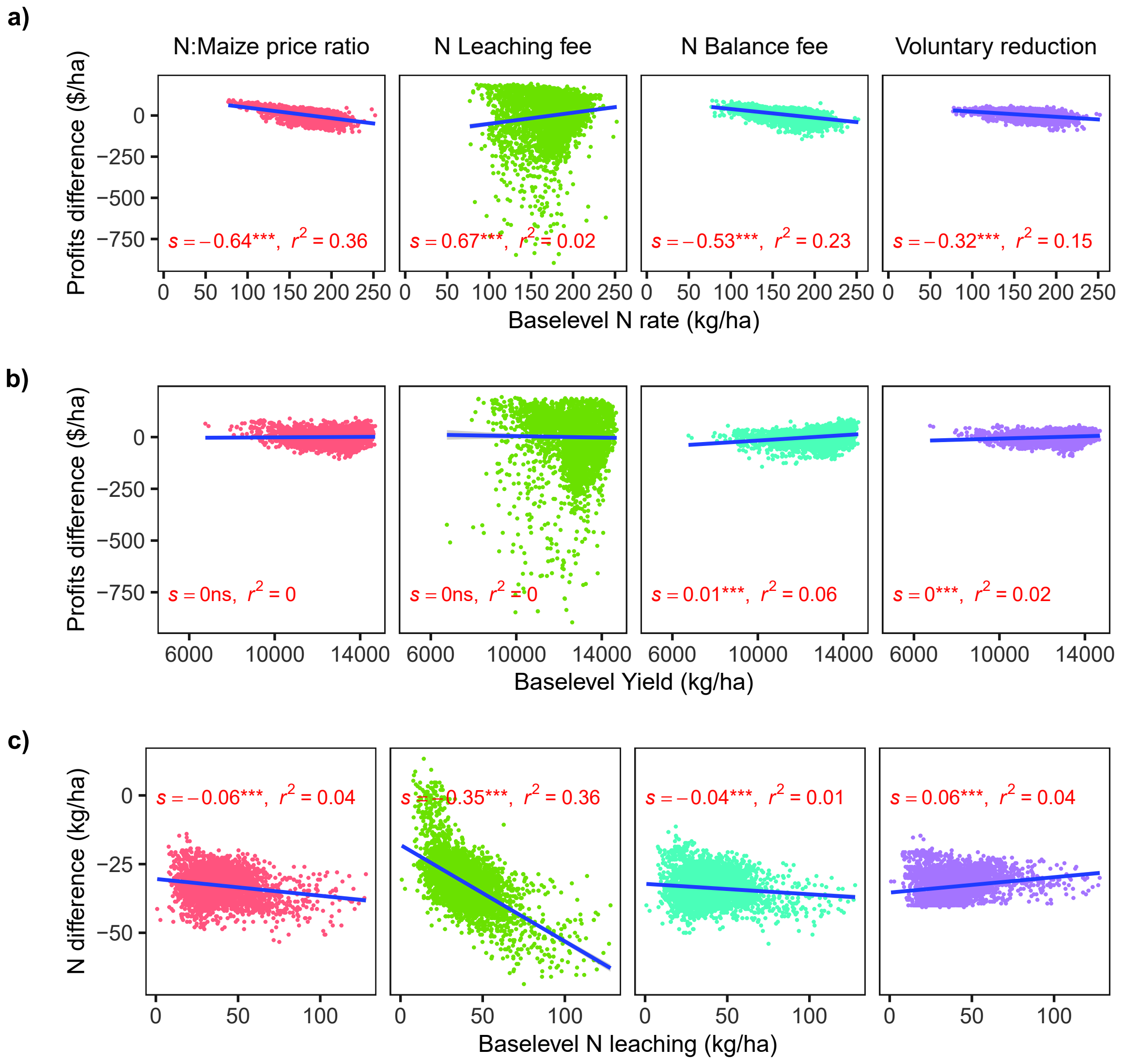

Figure S4: Field-level effects for the different policies: a) Profits difference relative to the base-level N rate. b) Profits difference relative to the base-level yield. c) N fertilizer difference relative to the base-level N leaching. Observations are at the field level, averaged across weather years. Linear regressions were fitted independently to each set of data, and report the estimate of the slope (s), test of significance (ns=non-significant; $*= p < 0.1$, $**= p < 0.05$; $***= p < 0.01$), and coefficient of determination (r^2)

References

Illinois-EPA (2015). “Illinois nutrient loss reduction strategy”. In: *Illinois Environ. Prot. Agency. http://www. epa. illinois. gov/Assets/iepa/water-quality/watershed-management/nlrs/nlrs-final-revised-083115. pdf (accessed 2 Feb. 2018).*
